# An alert tool to promote lung protective ventilation for possible acute respiratory distress syndrome

**DOI:** 10.1093/jamiaopen/ooac050

**Published:** 2022-07-08

**Authors:** Andrew J Knighton, Kathryn G Kuttler, Pallavi Ranade-Kharkar, Lauren Allen, Taylor Throne, Jason R Jacobs, Lori Carpenter, Carrie Winberg, Kyle Johnson, Neer Shrestha, Jeffrey P Ferraro, Doug Wolfe, Ithan D Peltan, Rajendu Srivastava, Colin K Grissom

**Affiliations:** Healthcare Delivery Institute, Intermountain Healthcare, Murray, Utah, USA; Digital Technology Services, Intermountain Healthcare, Salt Lake City, Utah, USA; Digital Technology Services, Intermountain Healthcare, Salt Lake City, Utah, USA; Healthcare Delivery Institute, Intermountain Healthcare, Murray, Utah, USA; Healthcare Delivery Institute, Intermountain Healthcare, Murray, Utah, USA; Division of Pulmonary and Critical Care Medicine Department of Medicine, Intermountain Medical Center, Murray, Utah, USA; Division of Pulmonary and Critical Care Medicine Department of Medicine, Intermountain Medical Center, Murray, Utah, USA; Division of Pulmonary and Critical Care Medicine Department of Medicine, Intermountain Medical Center, Murray, Utah, USA; Digital Technology Services, Intermountain Healthcare, Salt Lake City, Utah, USA; Digital Technology Services, Intermountain Healthcare, Salt Lake City, Utah, USA; Division of Epidemiology, Department of Internal Medicine, University of Utah School of Medicine, Salt Lake City, Utah, USA; Healthcare Delivery Institute, Intermountain Healthcare, Murray, Utah, USA; Division of Pulmonary and Critical Care Medicine Department of Medicine, Intermountain Medical Center, Murray, Utah, USA; Division of Pulmonary and Critical Care Medicine, Department of Internal Medicine, University of Utah School of Medicine, Salt Lake City, Utah, USA; Healthcare Delivery Institute, Intermountain Healthcare, Murray, Utah, USA; Department of Pediatrics, University of Utah School of Medicine, Salt Lake City, Utah, USA; Division of Pulmonary and Critical Care Medicine Department of Medicine, Intermountain Medical Center, Murray, Utah, USA; Division of Pulmonary and Critical Care Medicine, Department of Internal Medicine, University of Utah School of Medicine, Salt Lake City, Utah, USA

**Keywords:** clinical decision support systems, acute lung injury, acute respiratory distress syndrome, computerized ventilation protocols, lung protective ventilation

## Abstract

**Objective:**

Computer-aided decision tools may speed recognition of acute respiratory distress syndrome (ARDS) and promote consistent, timely treatment using lung-protective ventilation (LPV). This study evaluated implementation and service (process) outcomes with deployment and use of a clinical decision support (CDS) synchronous alert tool associated with existing computerized ventilator protocols and targeted patients with possible ARDS not receiving LPV.

**Materials and Methods:**

We performed an explanatory mixed methods study from December 2019 to November 2020 to evaluate CDS alert implementation outcomes across 13 intensive care units (ICU) in an integrated healthcare system with >4000 mechanically ventilated patients annually. We utilized quantitative methods to measure service outcomes including CDS alert tool utilization, accuracy, and implementation effectiveness. Attitudes regarding the appropriateness and acceptability of the CDS tool were assessed via an electronic field survey of physicians and advanced practice providers.

**Results:**

Thirty-eight percent of study encounters had at least one episode of LPV nonadherence. Addition of LPV treatment detection logic prevented an estimated 1812 alert messages (41%) over use of disease detection logic alone. Forty-eight percent of alert recommendations were implemented within 2 h. Alert accuracy was estimated at 63% when compared to gold standard ARDS adjudication, with sensitivity of 85% and positive predictive value of 62%. Fifty-seven percent of survey respondents observed one or more benefits associated with the alert.

**Conclusion:**

Introduction of a CDS alert tool based upon ARDS risk factors and integrated with computerized ventilator protocol instructions increased visibility to gaps in LPV use and promoted increased adherence to LPV.

## INTRODUCTION

Acute respiratory distress syndrome (ARDS) occurs when an acute lung injury (eg, pneumonia or COVID-19) causes bilateral noncardiogenic pulmonary edema and hypoxemic respiratory failure that requires invasive mechanical ventilation. An international study in 50 countries found that 10% of patients admitted to the intensive care unit (ICU) and 23% of mechanically ventilated patients had ARDS.^1^ Based upon COVID-19 data published by the Centers for Disease Control and Prevention (CDC), 20%–42% of hospitalized patients and 67%–85% of patients admitted to the ICU will develop ARDS.^2^ Lung-protective ventilation (LPV) is a protocol that combines low tidal volume ventilation (LTVV) with cotitration of oxygen delivery and the positive end expiratory pressure (PEEP) that improves outcomes for patients with ARDS^3^^,^^4^ and is recommended by the American Thoracic Society (ATS) Clinical Practice Guidelines^5^^,^^6^ and other recent reviews.^7^^,^^8^

At our institution, we have introduced computerized ventilator protocols using computable biomedical knowledge and patient-specific data to support the treatment of patients diagnosed with ARDS.^9–14^ While underrecognition of ARDS remains a challenge for clinicians,^15–17^ improved recognition may not be sufficient to drive increased use of LPV.^18^^,^^19^ Disease detection systems or “sniffer systems” have been proposed to improve the time to diagnosis of this syndrome and to encourage more timely treatment using LPV largely in academic settings with varied levels of sensitivity and positive predictive value.^20–22^ Given well-published concerns regarding alert fatigue in the ICU and its potential contribution to adverse patient outcomes,^23^ including LPV treatment detection logic following disease detection logic could reduce the incidence of unnecessary alerts where LPV treatment is already occurring.^24^^,^^25^

## OBJECTIVE

The purpose of this study was to evaluate implementation and service outcomes^26^ associated with deployment and use of a clinical decision support (CDS) synchronous alert tool (“the CDS alert tool”) targeting patients with possible ARDS but not receiving LPV. In the context of implementation studies, service outcomes differ from clinical treatment outcomes by focusing on the impact of the intervention on how the clinicians and related work processes are impacted and not its downstream effects on end customer or patient outcomes, which is measured separately. This CDS alert tool was designed to improve service outcomes by increasing visibility to nonadherent LPV practices, including adoption of related evidence-based computerized ventilator protocols and adherence to specific protocol instructions; promoting clinician behavior change; and minimizing unnecessary clinician alerts for patients already receiving LPV care.

## MATERIALS AND METHODS

We performed an explanatory, sequential mixed methods study (1) to measure CDS alert tool utilization, influence on clinician behavior and timeliness and (2) to understand CDS alert tool appropriateness and acceptability.^27^^,^^28^ The research protocol was approved by the Intermountain Healthcare Institutional Review Board (IRB No. 1050867). We adhered to published best practices for reporting of mixed methods studies.^29^ The study was conducted under a 2-year U-01 planning grant with the National Heart, Lung and Blood Institute (No. 1U01HL143505-01) and in conjunction with a pilot hybrid implementation-effectiveness trial (NCT03984175) designed to increase adherence to LPV in ARDS patients.

### Implementation context

During the study period, Intermountain Healthcare (Intermountain) operated 17 ICUs within 13 hospitals as part of an integrated, 24-hospital health system that included rural hospitals, tertiary care centers, and a children’s hospital. Across the system, over 4000 adult patients received invasive mechanical ventilation annually. Approximately 20% of these ventilated patients had ARDS.^1^^,^^30^ To improve the use of LPV in mechanically ventilated patients, computerized ventilator protocols making use of open-loop CDS reside within the electronic health record (EHR). Once the physician orders the computerized ventilator protocols, a clinician (usually a respiratory therapist) receives proposed ventilator setting change instructions from the computerized ventilator protocols but makes the final clinical determination regarding the setting changes. The computerized ventilator protocols integrate 4 different open-loop ventilator subprotocols: a LTVV protocol managing tidal volume and respiratory rate; an oxygenation protocol managing FiO_2_ and PEEP to target PaO2 of 55–68 mmHg (with options for normal or high PEEP strategies); a weaning assessment; and a spontaneous breathing protocol. The computerized ventilator subprotocols are deployed across all adult ICU sites using a single enterprise EHR platform (Cerner Corporation, Kansas City, MO, USA).

### Intervention

**Figure 1.**
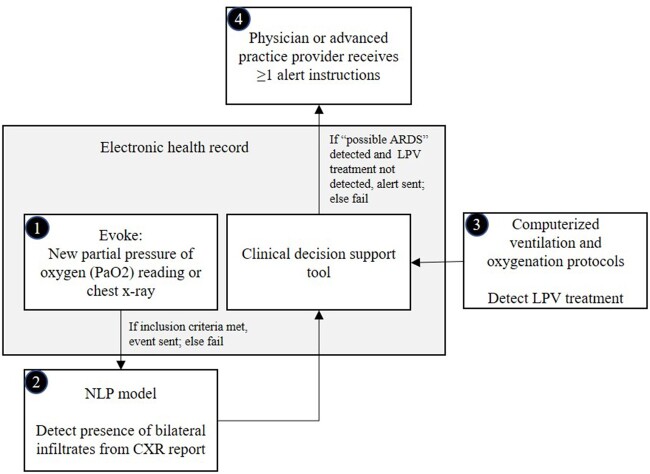
Data flow architecture for the clinical decision support (CDS) synchronous alert tool. (1) Patient care generates new partial pressure of oxygen (PaO2) reading or new chest X-ray trigger event. Trigger events meeting inclusion criteria are sent to a natural language processing (NLP) model; else fail. (2) The NLP model detects the presence of bilateral infiltrates per the chest radiographic report. All positive and negative events are sent to the electronic health record CDS tool. (3) If “possible ARDS” is detected, the clinical decision tool logic receives data from the computerized ventilation and oxygenation protocols confirming appropriate patient treatment for ARDS using lung protective ventilation (LPV). (4) If “possible ARDS” is detected and LPV treatment is not detected, the physician or advanced practice provider receives ≥1 alerts with ≥1 instructions; else fail. New patient biomedical data may evoke a new cycle.

The CDS alert tool was developed by members of the Center of Excellence in Critical Care Implementation Research at Intermountain including critical care clinicians, informaticists, and health services researchers.^31^ The rules for identifying the presence of risk factors for ARDS were based upon 2 components of the Berlin consensus diagnosis criteria: (1) presence of bilateral infiltrates on a chest X-ray and (2) the ratio of the arterial partial pressure of oxygen (PaO_2_ in mmHg) from an arterial blood gas divided by the fraction of inspired oxygen (FiO_2_), or altitude-adjusted P/F ratio <255.^32^ The CDS alert tool data flow architecture is depicted in Figure 1 and principal data components are summarized in Table 1.

**Table 1.** Clinical decision support system functions and logic

Function	Result generated	Logic
Trigger	Trigger event generated	(1) PaO_2_ is charted; or (2) a new chest X-ray.
		Inclusion criteria: Patient ≥18 years Ventilator mode charted <7 days ago Altitude-adjusted P/F ratio ≤255 Patient was intubated within the last 4 days
		Exclusion criteria: In a PICU On palliative care Attending physician is a cardiothoracic surgeon On ventricular assist device or mechanical circulatory support Breathing spontaneously without positive pressure ventilation via a T-piece or tracheostomy mask Presence of a pulmonary artery catheter
Disease detection using NLP	Possible ARDS event generated	NLP identification of bilateral infiltrates on chest radiograph report
LPV treatment detection	Clinician alert	Possible ARDS event where (1) altitude-adjusted P/F ratio ≤255 and (2) current patient treatment noncompliant with ventilation and oxygenation protocols

*Note*: If a trigger event is positive for possible ARDS and LPV treatment is not detected, the CDS tool will evoke one or more clinician alerts

EHR: electronic health record; PICU: pediatric intensive care unit; NLP: natural language processing; PaO_2_: partial pressure of oxygen; P/F ratio: ratio of the PaO_2_ (arterial oxygen partial pressure obtained from an arterial blood gas) to the FiO_2_ (fraction of inspired oxygen); CDS: clinical decision support; LPV: lung protective ventilation; ARDS: acute respiratory distress syndrome.

A clinician alert is generated using a 3-step logic: (1) trigger events are detected in the EHR as outlined in Table 1; (2) presence of possible ARDS is detected using an NLP algorithm to identify the presence of bilateral infiltrates on the patient’s chest radiograph report; and (3) if possible ARDS is present and the patient treatment is noncompliant with oxygenation and ventilation protocols, the clinician receives one or more alert messages with one or more recommendations. Clinicians receiving the alert are limited to treating physicians and advanced practice providers caring for the patient.

The CDS alert tool was defined with 39 unique alert code types—with each alert code type representing a combination of one or more unique alert messages and with one or more recommendations (Supplementary Table S1). The alert message displays the radiographic and physiologic evidence for evoking the alert and specifies ventilator settings causing initial LPV nonadherence. The clinician is informed of the nonadherence via an alert message when they open the patient chart in the EHR. The clinician can then: (1) order the appropriate protocol or act on specific protocol instructions; (2) indicate the patient does not have ARDS; or (3) provide a reason the protocol is contraindicated. If the clinician indicates the patient does not have ARDS or selects a contraindication, alerts are permanently suppressed for all clinicians associated with that patient and will no longer evoke. To document contraindications, clinicians can select elevated intracranial pressure, status asthmaticus, or severe chronic obstructive pulmonary disease from a drop-down list or document another reason in a free-text field. The clinician may be reminded again when they close the patient’s chart if there are certain alert recommendations that have not been addressed since opening the patient chart. To reduce the risk of alert fatigue, the alert only evokes once per shift (every 16 h) per clinician. New patient-specific biomedical data can initiate a new cycle.

The CDS alert tool was developed within the enterprise EHR platform Cerner Millennium using an event-driven rules-based (Boolean logic) CDS solution. The alert was coded using a full-featured, fourth-generation programming language.^33^ The Cerner Command Language (CCL), a structured query language, was used to query the Cerner Millennium database.^33^ HTML and JavaScript were used to create a more interactive user interface.

### Study populations

The quantitative study population included all patient encounters where a patient was admitted to 1 of 13 Intermountain hospitals with an ICU from December 2019 to November 2020 where the CDS alert tool was implemented and for whom at least one trigger event was evoked during the encounter. Encounters for patients hospitalized after brain death for organ donation, hospice/comfort care patients, patients <18 years old, and patients admitted under a cardiothoracic surgeon were excluded. A patient could have more than 1 encounter. For qualitative outcomes, the eligible survey population included all clinicians (physicians and advanced practice providers) with a patient having at least one possible ARDS event generated (whether or not event was adherent to LPV treatment protocols) and who received care at 1 of 6 Intermountain hospitals containing at least one ICU from October 1 to November 30, 2020.

### Data collection

For quantitative outcomes, we obtained trigger events and possible ARDS events detected (including events compliant and noncompliant with LPV treatment) from the Intermountain electronic data warehouse. For qualitative outcomes, 2 web-based surveys were developed targeting individual clinicians with one or more possible ARDS events either resulting in (1) ≥1 alert(s); or (2) no alerts given existing compliance with LPV treatment protocols. Survey questions were developed and adapted from the Unified Theory of Acceptance and Use of Technology (UTAUT)^34^ with Likert-style categorical responses in 1 of 5 categories (strongly disagree, disagree, indifferent, agree, strongly agree). Survey questions were validated with 2 intensivist subject matter experts using the think-aloud method of cognitive interviewing.^35^ Prior to the distribution of surveys, our healthcare system’s senior medical director of critical care prenotified potential survey participants (all physicians and advanced practice providers with a primary work assignment in an ICU who regularly manage mechanically ventilated patients) via an email that described the purpose of the study and encouraged participation. Surveys were distributed via an individualized email to the clinician 4 days after the possible ARDS detection event (regardless of whether an alert was actually sent). Up to 3 subsequent email reminders to complete the survey were sent if the survey was not completed (day 5, 6, and 9 after the positive prescreen event). The survey interface required respondents to answer every question before survey submission, resulting in no partial responses or missing data. Respondents were limited to a single survey response. The final format and content for both surveys are included in Supplementary Appendices S1 and S2. Study data were collected and managed using the Research Electronic Data Capture system (REDCap, Vanderbilt University, Nashville, TN, USA).^36^^,^^37^

### Study outcomes

Study outcomes were classified using standards for implementation research.^26^ Implementation outcomes included appropriateness, discriminatory power, and acceptability of the CDS alert tool and its accuracy. In implementation research, service outcomes are derived from the Institute of Medicine report on quality improvement aims in healthcare.^26^ Service outcomes were used to evaluate increased visibility to nonadherent practices, including implementation effectiveness in adopting the computerized ventilator protocols and adherence to specific protocol instructions; clinician behavior change; and minimizing unnecessary clinician alerts for patients already receiving evidence-based care. Patient or client outcomes, including clinical effectiveness outcomes associated with increased adherence to the evidence-based guidelines, were not measured (Supplementary Table S2).

#### Implementation outcomes

To evaluate appropriateness, we measured the percentage of survey respondents who (1) indicated that the use of alerts fits with the way they like to work and (2) would have preferred to receive an alert despite being LPV-adherent. To measure CDS tool discriminatory power in detecting possible ARDS, including sensitivity and positive predictive value, we investigated a group of intubated patients admitted December 2019 to February 2020 for whom gold-standard adjudication of ARDS was available and generated a contingency matrix and summary statistics including a receiver operating characteristics (ROC) curve. We then estimated the area under ROC curve (AUROC) to measure the probability that a pair of positive or negative CDS tool results would be accurately categorized as having or not having ARDS. To evaluate acceptability of the CDS alert tool, we measured the percentage of survey participants who identified one or more benefits associated with the receipt of an alert.

#### Service outcomes

We evaluated several measures of implementation effectiveness, defined as avoiding the over and under use of resources and services.^26^ We generated 2 measures of guideline adherence. The initial guideline nonadherence measure counted the number of encounters during the study period where possible ARDS was detected and patient treatment was not adherent to the clinical guideline. This measure provides transparency into how often individual LPV guidelines were being followed prior to any alert being sent. Initial guideline nonadherence was measured by dividing the number of patient encounters with ≥1 possible ARDS events where LPV treatment was not detected by the number of patient encounters with ≥1 trigger events.

The percentage of time a specific alert recommendation was followed, or alert recommendation adherence, was calculated by dividing the individual recommendations (an alert message may have ≥1 recommendations) that were followed by the total alert recommendations sent to clinicians. A recommendation was classified as followed if there was a change in the underlying treatment management consistent with the individual recommendation usually within a 2-h period following receipt of the alert message (Supplementary Table S1).

The number of possible disease detection alerts prevented as a result of introducing logic to assess the status of current LPV treatment was calculated by taking the difference between the number of events where possible ARDS was detected (whether or not LPV treatment was detected) and multiplying the difference by the mean number of alert messages sent per possible ARDS events where LPV treatment was not detected at each site.

Timeliness was measured by dividing the number of clinicians reporting that the alert decreased time to detection of ARDS by the total survey participants receiving the alert.

### Statistical analysis

Survey responses were grouped into 2 binary categories of agreement (agree or strongly agree) or disagreement (disagree or strongly disagree) with “indifferent” grouped along with 1 of these 2 categories based on the intent of the question as noted in the results. Responses were further analyzed by respondent attributes: clinician type (physician, nurse practitioner, and physician assistant), sex (female, male), years of clinical experience (0–5, 6–10, 11–15, more than 15), and ICU hospital site where patient was receiving care (6 locations). Between-group comparisons based upon demographic factors were made using Pearson’s chi-square test or Fisher’s exact test. A *P* value <.05 was considered significant. Presentation of survey results and contingency matrix summary statistics were calculated using binomial exact 95% confidence intervals (CI). For AUROC, we utilized a nonparametric method with the standard error estimated using the Hanley–McNeil algorithm.^38^ All statistical analyses were conducted in Stata version 13 (StataCorp LP, College Station, TX, USA).

## RESULTS

During the study period, at least one trigger event occurred during 1041 patient encounters involving 1016 unique patients (Table 2). As defined earlier, there were 1553 trigger events, 775 events where possible ARDS was detected and 455 events where possible ARDS was detected and LPV treatment was not detected. A total of 158 clinicians had possible ARDS detection events where LPV treatment was not detected during the survey period and 53 responded to 1 of the 2 surveys (response rate 34%). Survey response rates did not vary significantly based upon clinician role, sex or whether possible ARDS was detected and either the clinician received or did not receive the alert because the patient was compliant with treatment guidelines. Site level response rates varied significantly across participating sites (Supplementary Table S3).

**Table 2.** Clinical decision support synchronous alert tool performance data by hospital site (*n* = 1041 encounters)

	Patient encounters with ≥1 trigger events meeting inclusion criteria	Initial detection of guideline nonadherence	Total alerts sent	Total alerts sent per possible ARDS event and treatment is nonadherent	Percentage of potential alerts prevented through assessing guideline adherence before sending alert
Total	1041	38%	2876	6.3	41
Tertiary hospital					
A	356	40%	1718	10.1	36
B	235	28%	225	3.0	59
C	220	39%	475	4.7	40
D	150	43%	243	3.4	28
E	51	59%	161	5.3	33
Community hospital					
F	4	50%	43	21.5	33
G	9	22%	6	3.0	67
H	5	40%	2	1.0	33
I	5	0%	0	NA	0
J	4	50%	43	21.5	33
K	3	33%	3	3.0	0
L	2	0%	0	NA	0
M	1	0%	0	NA	0
Tertiary hospitals only	1012	39%	2822	6.3	41
Community hospitals only	29	24%	54	7.7	53

### Implementation effectiveness

Thirty-eight percent of study encounters had at least one episode of initial guideline nonadherence; the proportion varied by site (range: 22%–59%). The 455 events with possible ARDS where LPV treatment was not detected evoked 2876 alert messages, or 6.3 alert messages per event. Overall, use of the possible ARDS detection event where LPV treatment was not detected versus possible ARDS detection event alone to evoke clinician-facing alerts prevented an estimated 1812 alert messages (41%) varying by site (Table 2). The 2876 individual alert messages sent contained 3281 individual recommendations (some alert code types included more than 1 recommendation) grouped into 2 general categories: (1) recommendations promoting adoption of the computerized ventilator protocols (34% of alert recommendations) or (2) specific ventilation recommendations to those already using the computerized ventilator protocol (66% of alert recommendations). Overall, 48% of the recommendations were followed within the defined adherence timeframe (see Supplementary Table S1), including 65% of recommendations for patients already ordered for ventilator protocols, but only 14% of alerts prompting adoption of the computerized ventilator protocols (proportion difference: 0.51 [95% CI: 0.48–0.54]; *P* < .001). Considerable variation existed in the percentage of recommendations implemented by individual recommendation type (Table 3).

**Table 3.** Clinician adherence to clinical decision support synchronous alert tool recommendations (*n* = 3281 alert recommendations)

Alert recommendation	Individual recommendation count	Percent total instructions	Recommendation followed count	Percent adherent
Followed recommendations to adopt computerized ventilator protocols				
Computerized ventilation and oxygenation protocols not ordered	608	18.5	36	5.9
Use of CPAP/PS is inappropriate because PEEP > 10, or FiO_2_ > 50%, or PS > 15	156	4.8	64	41.0
Consider changing to volume control mode per the ventilation protocol	156	4.8	46	29.5
Standard ventilator mode not in use	117	3.6	10	8.5
Computerized ventilation protocol not ordered	49	1.5	0	0.0
Health system compliant ventilator mode not in use	17	0.5	0	0.0
Computerized oxygenation protocol not ordered	16	0.5	0	0.0
Subtotal	1119	34.1	156	13.9
Followed specific computerized ventilator protocol recommendations				
Current inappropriate PEEP/FiO_2_ combination	1918	58.5	1371	71.5
Tidal volume is too large	244	7.4	39	16.0
Subtotal	2162	65.9	1410	65.2
Total	3281	100.0	1566	47.7

*Note*: These results are grouped by individual instruction. A given alert may have more than 1 instruction. Overall, 48% of the alert recommendations were followed within the defined adherence timeframe. Considerable variation existed in the percentage of recommendations implemented by individual recommendation type.

CPAP: continuous positive airway pressure; PS: pressure support; PEEP: positive end-expiratory pressure; FiO_2_: frequency of inspired oxygen.

### Acceptability

Fifty-seven percent of survey respondents identified one or more potential benefits associated with use or potential use of the alert or expressed a preference to have received the alert message (57% [95% CI: 43%–70%]) (Table 4). Among the 22 survey respondents who had received an alert message, 23% reported the alert message raised their awareness of risk factors for ARDS; 45% agreed the alert message provided diagnostic information; and 32% reported the alert message provided useful information for making ventilation management decisions. No clinicians receiving the alert message indicated that it shortened the time to diagnosis of ARDS. Among surveyed respondents not receiving an alert message (*n* = 31), 40% felt that receiving the alert message would have improved their ability to deliver care for patients with ARDS even when mechanical ventilation settings were optimal (45%) and that not receiving the alert message impacted their ability to make decisions about ventilation strategies (42%).

**Table 4.** The results of 2 web-based surveys to evaluate clinician attitudes regarding a clinical decision support synchronous alert tool (*n* = 53 total survey participants)

Survey statements	% Agree or strongly agree (except as noted)		95% CI lower limit	95% CI upper limit
All participants (*n* = 53)				
Clinicians who identified at least one more potential benefits associated with use or potential use of the alert or who expressed a preference to have received the alert	57		43%	70%
Clinicians receiving an alert because they were not adherent to lung protective ventilation (*n* = 22)				
Using an automated alert in iCentra fits with the way I like to work	73^a^		54%	91%
The alert raised my awareness of risk factors associated with ARDS	23		5%	40%
The alert provided diagnostic information for ARDS	45		25%	66%
The alert provided useful information in making ventilation strategy decisions (mode, tidal volume, PEEP/FiO_2_ combination, CPAP/PS, etc.).	32		12%	51%
The alert shortened the time to diagnosis of ARDS.	0		N/A	N/A
Acting upon the information in the alert improved the delivery of patient care.	36		16%	56%
The clinician identified at least one or more benefits from above associated with use of the alert.	50		29%	71%
Clinicians not receiving an alert because they were adherent to lung protective ventilation (*n* = 31)				
Using an automated alert in iCentra fits with the way I like to work.	84^a^		71%	97%
Receiving the alert would improve my ability to deliver care for patients with ARDS, even when mechanical ventilation settings are optimal.	45		28%	63%
Not receiving the alert impacted my ability to diagnose ARDS.	3		0%	9%
Not receiving the alert impacted my ability to make decisions about ventilation strategies (mode, tidal volume, PEEP/FiO_2_ combination, CPAP/PS, etc.).	42		25%	59%
I would have preferred to have received the alert.	35		19%	52%
The clinician identified at least one or more potential benefits from above associated with potential use of the alert or expressed preference to have received the alert.	61		44%	78%

*Note*: The 2 surveys targeted individual clinicians with one or more encounters where possible ARDS was detected resulting in (1) ≥1 alert(s); or (2) no additional action given existing adherence to lung protective ventilation.

ARDS: acute respiratory distress syndrome; CPAP: continuous positive airway pressure; PS: pressure support; PEEP: positive end-expiratory pressure; FiO_2_: frequency of inspired oxygen; iCentra: Intermountain Healthcare’s branded name for Cerner’s Millennium platform.

a Includes those who were indifferent to using alerts. Significant using 2-tailed test of significance at *P* < .05.

### Appropriateness

Overall, a majority (79% [95% CI: 0.68–0.90]) of clinician survey respondents strongly agreed, agreed, or were indifferent to the statement that generally using an automated alert in the EHR fits with the way they like to work (when limiting to those who agree or strongly agree solely: 68% [95% CI: 53%–84%]) (Supplementary Table S3). Sixty-four percent of survey respondents were from ICUs with a long history of using CDS tools in critical care, with the highest levels of agreement with tool use (89%) among clinicians with more than 15 years of experience. Thirty-five percent of survey participants who had a possible ARDS detection event (but did not receive the alert because their patient was compliant with LPV) agreed or strongly agreed they would have preferred to receive the alert message. Among 73 intubated patients, the AUROC of the CDS alert tool was 0.62 (95% CI: 0.47–0.74), with a sensitivity of 0.87 (95% CI: 0.73–0.96), a false positive rate of 0.66 (95% CI: 0.45–0.80), and a positive predictive value of 0.62 (95% CI: 0.48–0.75) (Supplementary Table S4). While the false-positive rate was 0.66, only 38% of false positive events evoked an alert.

## DISCUSSION

Introduction of a CDS alert tool embedded within the EHR and integrated with computerized ventilator protocol recommendations increased visibility to gaps in adherence to LPV among patients with ARDS. Limiting alerts to situations where possible ARDS was detected and LPV treatment was not detected prevented 41% of possible disease alerts. The alert was more successful in influencing adherence to evidence-based recommendations among patients for whom the ventilator protocols were already ordered than in increasing adoption of the protocols.

Initial guideline nonadherence was detected in 38% of patient encounters. Most recommendations (66%) were associated with presence of inappropriate PEEP/FiO_2_ combinations. Our findings are consistent with other studies noting that even when clinicians intend to use LPV strategies and think they are compliant, implementation of LPV remains variable.^19^^,^^39^ The presence of nonadherence even when computerized ventilator protocols are present highlights the operational complexity of deploying optimal ventilator management. A multilevel intervention such as ours combining protocolized open-loop CDS with best-practice alerts can help overcome barriers such as ARDS under recognition, resource constraints, and the complex nature of the RT/clinician dynamic.^15^

Efforts at developing computerized disease detection systems should consider determining not only the presence of a disease, but whether active treatment is already underway. In CDS systems involving disease detection alone, diagnostic alert messages may increase alert fatigue without improving care.^40^ Use of CDS to promote guideline adherence in conjunction with a disease detection system has been shown in hospitalized patients with risk of other conditions.^41–43^ The use of an alert conditioned on both possible disease presence and detection of LPV treatment successfully prevented an estimated 41% of alerts, reducing the level of potential signal noise in the environment and overalerting using disease detection alone. Reasons for the relatively high rates of reduction can be attributed to the already high use of the existing computerized ventilator protocols, as well as an accepted system-level policy that LPV is safe to initiate using the computerized ventilator protocols for most mechanically ventilated patients.

The alert recommendations appeared most successful in influencing adherence to evidence-based instructions among patients for whom computerized ventilator protocols were already ordered. In these circumstances, clinicians have already adopted the computerized ventilation protocols. The recommendations refined their existing use of an LPV strategy (adjusting PEEP/FiO_2_ combinations or tidal volume, for example). In contrast, the alert appeared less successful in prompting the ordering of the computerized ventilator protocols and the initiation of LPV strategy when not present. Increasing adoption of the computerized ventilator protocols among this subset of clinicians presents a more complex set of behavior changes that may require a broader, multimodal implementation approach.^15^

The percentage of clinicians who felt that alert use generally fits with the way they like to work was almost 80% and did not highlight clinician resistance described more generally to alerts.^44–46^ Our institution has developed a process for computerized ventilator protocol development that uses informatics and development engineering teams collaborating with clinicians.^11^^,^^12^ The use of a methodologically rigorous approach responsive to user needs and perceptions is associated with higher user acceptance.^47–49^ The fact that most providers were highly experienced intensivists and worked in ICUs with a long history of using CDS tools in critical care may have improved alert acceptance. Clinicians’ ability to diagnose ARDS accurately and timely could not be independently verified but our results suggest that clinicians felt confident in their ability to rapidly detect and treat ARDS.

Our CDS alert tool demonstrated a high sensitivity and modest, but adequate positive predictive value. In a systematic review of electronic detection systems or “sniffer tools” developed to detect ARDS, Wayne et al,^22^ found a wide range of sensitivities (43%–98%) and positive predictive values (26%–90%) across 9 studies. Given the tool’s dual goals to increase awareness of possible ARDS and identify deviations from lung-protective mechanical ventilation among these patients, the performance characteristics of this CDS alert tool appeared sufficient given the low risk of harm and the increasingly common practice of treating non-ARDS patients with LPV.^50^^,51^ While the proportion of false positive trigger events was relatively high (.66), only 38% of these ultimately evoked an alert given existing adherence to LPV, lowering the impact of false positive trigger results on front-line caregivers.

Strengths of this implementation study include its comprehensive approach to evaluating implementation of the CDS tool in a nonacademic, community-based health system. This observational implementation study was not designed to generate causal inferences regarding whether the use of the CDS alert tool was superior to the lack of an alert or to disease detection alerts alone in promoting behavior change. The CDS tool only utilized 2 of 4 Berlin criteria in identifying patients with possible ARDS and was not designed as a complete disease detection system. While the trigger criteria were developed a priori rather than derived from patient data, we employed a small dataset of patients for trigger event validation and our estimates of the CDS tool’s discriminatory power should be considered preliminary and requiring confirmation in both larger and external datasets. Though the alert was implemented across a diverse set of ICUs, they all belonged to a single health system using a single EHR and access to natural language processing input for imaging results. The broad generalizability of our findings to other health systems and EHRs requires future study.

## CONCLUSION

Introduction of a synchronous CDS alert tool based upon ARDS risk factors and integrated with computerized ventilation protocol recommendations increased visibility to gaps in adherence and promoted increased use of LPV. Efforts to develop ARDS disease detection systems should evaluate the use of alerts conditioned on the presence of evidence-based treatment to minimize alert fatigue.

## FUNDING

This work was supported by the National Heart, Lung and Blood Institute (Grant No. 1U01HL143505-01) and the National Center for Advancing Translational Sciences (Grant No. KL2TR002539) of the National Institutes of Health.

## AUTHOR CONTRIBUTIONS

AK, KK, PR, IP, RS, and CG contributed to the conception and design of this study. The remaining coauthors made specific contributions to data acquisition and analysis. All authors participated in the initial drafting and revisions of the work; approved the final version; and accept accountability for the overall integrity of the research process and the article.

## Supplementary Material

ooac050_Supplementary_Data
