# Supplementary material for: An alert tool to promote lung protective ventilation for possible acute respiratory distress syndrome
Source: JAMIA Open. 2022 Jul 8;5(2):ooac050. doi: 10.1093/jamiaopen/ooac050 (PMC9263532; doi:10.1093/jamiaopen/ooac050)
Supplement: ooac050_Supplementary_Data [file ooac050_supplementary_data.zip › Appendices_R1.docx]

**APPENDIX 1 – SURVEY – CLINCIANS WHO RECEIVED AN ALERT**


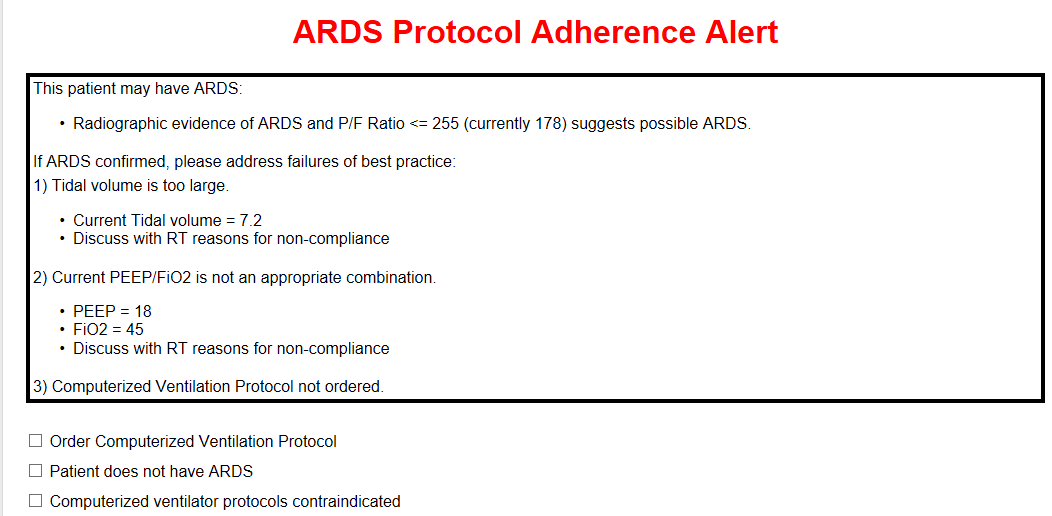


In 2020, an Intermountain Critical Care research team led by the healthcare system’s Senior Medical Director of Critical Care implemented an “exception-driven” acute respiratory distress syndrome (ARDS) Protocol Adherence Alert in Cerner that was designed to:

1. Identify patients with signs of ARDS based upon a qualifying P/F ratio (≤255) and natural language processing-based interpretation of a chest X-ray with bilateral infiltrates.
2. Inform clinicians that the patient is not receiving optimal mechanical ventilation settings for ARDS.

The purpose of this survey is to determine clinician attitudes regarding the usefulness of this ARDS Protocol Adherence Alert. Participation in this survey is voluntary. You may refuse to take part in the research or exit the survey at any time without penalty. By answering the questions and submitting your response, you are giving your consent to use of the anonymized survey results for research purposes.  During the past week, you had at least one patient that triggered a positive prescreening event in iCentra (based on P/F ratio and radiographic evidence of ARDS) and resulted in one or more ARDS Protocol Adherence Alerts similar to the image above. Please read the following statements regarding the ARDS Protocol Adherence Alert and indicate the extent to which you agree or disagree with each statement.

| Survey Statements |
| --- |
| 1. Using an automated alert in iCentra (Cerner) fits with the way I like to work. |
| 1. Do you remember recently seeing one or more ARDS Protocol Adherence Alerts? |
| 1. The ARDS Protocol Adherence Alert raised my awareness of risk factors associated with ARDS. |
| 1. The ARDS Protocol Adherence Alert provided diagnostic information for ARDS. |
| 1. The ARDS Protocol Adherence Alert provided useful information in making ventilation strategy decisions (mode, tidal volume, PEEP/FiO2 combination, CPAP/PS etc.). |
| 1. The ARDS Protocol Adherence Alert shortened the time to diagnosis of ARDS. |
| 1. Acting upon the information in the alert improved the delivery of patient care. |
| 1. How many years of clinical experience do you have (post-training)? |
| 1. 0-5 |
| 1. 6-10 |
| 1. 11-15 |
| 1. More than 15 |

**APPENDIX 2 – SURVEY – CLINICIANS WHO DID NOT RECEIVE AN ALERT**


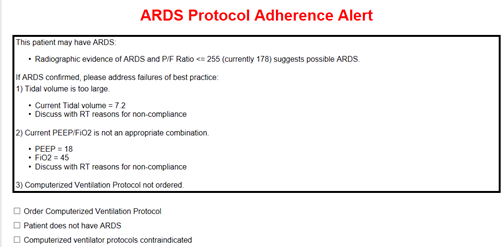


In 2020, an Intermountain Critical Care research team led by the healthcare system’s Senior Medical Director of Critical Care implemented an “exception-driven” acute respiratory distress syndrome (ARDS) Protocol Adherence Alert in Cerner that was designed to:

1. Identify patients with signs of ARDS based upon a qualifying P/F ratio (≤255) and natural language processing-based interpretation of a chest X-ray with bilateral infiltrates.
2. Inform clinicians that the patient is not receiving optimal mechanical ventilation settings for ARDS.

The purpose of this survey is to determine clinician attitudes regarding this ARDS Protocol Adherence Alert. Participation in this survey is voluntary. You may refuse to take part in the research or exit the survey at any time without penalty. By answering the questions and submitting your response, you are giving your consent to use the anonymized survey results for research purposes.

During the past week, you had at least one patient encounter that triggered a positive prescreening event in iCentra (based on P/F ratio and radiographic evidence of ARDS) but did not receive an alert because ventilation management strategies were appropriate.  Alerts are similar to the image above.

Please read the following statements regarding the ARDS Protocol Adherence Alert and indicate the extent to which you agree or disagree with each statement.

| Survey Statements |
| --- |
| 1. Using an automated alert in iCentra (Cerner) fits with the way I like to work. |
| 1. Receiving the ARDS Protocol Adherence Alert would improve my ability to deliver care for patients with ARDS, even when mechanical ventilation settings are optimal. |
| 1. Not receiving the alert did not impact my ability to diagnose ARDS. |
| 1. Not receiving the alert did not impact my ability to make decisions about ventilation strategies (mode, tidal volume, PEEP/FiO2 combination, CPAP/PS etc.). |
| 1. I would have preferred to have received an ARDS Protocol Adherence Alert. |
| 1. How many years of clinical experience do you have (post-training)? |
| 1. 0-5 |
| 1. 6-10 |
| 1. 11-15 |
| 1. More than 15 |
