# Supplementary material for: An alert tool to promote lung protective ventilation for possible acute respiratory distress syndrome
Source: JAMIA Open. 2022 Jul 8;5(2):ooac050. doi: 10.1093/jamiaopen/ooac050 (PMC9263532; doi:10.1093/jamiaopen/ooac050)
Supplement: ooac050_Supplementary_Data [file ooac050_supplementary_data.zip › supplemental_table_1_R1.docx]

| Supplemental Table 1. Possible alert messages and related recommendations | | | |
| --- | --- | --- | --- |
| Alert Message | Recommendation | Order Button | Evidence Recommendation Followed |
| Intermountain compliant ventilator mode not in use. Current Vent Mode = APRV | Discuss with RT current ventilation strategy and determine if changes are appropriate | No | Ventilator mode changed to MMV, VC, PRVC, or VC+ within 2 hours |
| Computerized Ventilation and Oxygenation Protocols not Ordered. | Order computerized ventilation and oxygenation protocols | Yes | Ordered oxygenation protocol AND ventilation protocol within 2 hours |
| Computerized Ventilation Protocol not Ordered. | Order computerized ventilation protocol | Yes | Ordered ventilation protocol within 2 hours |
| Computerized Oxygenation Protocol not ordered | Order computerized oxygenation protocol. | Yes | Ordered oxygenation protocol within 2 hours |
| Tidal volume is too large. Current Tidal volume = xx | Discuss with RT current ventilation strategy and determine if changes are appropriate | No | Tidal volume set to ≤6.5ml/kg within 2 hours |
| Current inappropriate PEEP/FiO2 combination. PEEP = xx; FiO2 = yy | Discuss with RT current ventilation strategy and determine if changes are appropriate. Order Computerized Oxygenation Protocol. | Yes | Use of [appropriate FiO2 and PEEP combination] OR [appropriate CPAP/PS] up to six hours after alert fires |
| Standard ventilator mode not in use. Ventilation mode = xx | Discuss with RT current ventilation strategy and determine if changes are appropriate. Order Computerized Ventilation and Oxygenation Protocols | Yes | Ventilator mode changed to MMV, VC, PRVC, or VC+ within 2 hours |
| Use of CPAP/PS is inappropriate because PEEP>10, or FiO2 > 50%, or PS > 15. | Discuss with RT current ventilation strategy and determine if changes are appropriate. Order all components in the Computerized Ventilator Protocol Orderset | Yes | [Change to FiO2 ≤50% and PEEP ≤10 and PS ≤15] OR [change to MMV, VC, PRVC, or VC+] within 2 hours |
| Consider changing to volume control mode per the ventilation protocol. |  |  | Ventilator mode changed to MMV, VC, PRVC, or VC+ within 2 hours |
| *Note that an alert message may be evoked individually or in combination with other alert messages and related instructions. APRV: Airway Pressure Release Ventilation; MMV: Mandatory Minute Ventilation; VC: Volume Control; VC+: Volume Control Plus; PRVC: Pressure-Regulated Volume Control; CPAP: Continuous Positive Airway Pressure; PS: Pressure Support; PEEP: Positive End-Expiratory Pressure; FiO2: Frequency of Inspired Oxygen; RT: Respiratory Therapist* | | | |
