# Supplementary material for: An alert tool to promote lung protective ventilation for possible acute respiratory distress syndrome
Source: JAMIA Open. 2022 Jul 8;5(2):ooac050. doi: 10.1093/jamiaopen/ooac050 (PMC9263532; doi:10.1093/jamiaopen/ooac050)
Supplement: ooac050_Supplementary_Data [file ooac050_supplementary_data.zip › supplemental_table_2_R1.docx]

| Supplemental Table 2. Study outcome descriptions and measure calculations | |
| --- | --- |
| Measurement Category | Description and measure calculation |
| Implementation Outcomes | |
| Appropriateness – Was the use of the CDS alert tool (including the alert design) an appropriate mechanism for promoting (1) adoption of the computerized ventilation protocols and (2) adherence to specific computerized ventilator protocol recommendations? | Percentage of clinicians who indicate that, in general, the use of alerts fits with way they like to work |
|  | Percentage of survey participants who still would have preferred to have received an alert message despite being adherent to LPV |
|  | Estimated accuracy of the CDS alert tool in identifying possible ARDS |
| Acceptability – Was the CDS tool agreeable, palatable or satisfactory as a mechanism for improving evidence-based care? | Percentage of survey participants who identified one or more benefits associated with the receipt of an alert message |
| Service (Process) Outcomes | |
| Implementation Effectiveness – Was this CDS tool as designed effective in achieving its goals – namely identifying non-adherence to evidence-based care and promoting increased adoption of the computerized ventilation protocols and adherence to specific computerized ventilation protocol instructions, while minimizing the number of alerts sent? | Initial treatment guideline non-adherence - number patient encounters with ≥1 qualifying prescreen events divided by the number of patient encounters with ≥1 trigger events. |
|  | Percentage of potential alert messages avoided by site given LPV adherence detection was calculated by dividing the estimated number of alert messages eliminated at each site by the total possible alert messages at each site [# of possible ARDS events * mean number of alert messages per possible ARDS events where LPV treatment was detected]. [Number of eliminated alert messages = (# of events where possible ARDS detected - # of events where possible ARDS detected and LPV treatment not detected)events) * mean number of alert messages per event where possible ARDS is detected and LPV treatment is not detected ]. |
|  | The percentage of time a specific alert recommendation was followed by calculated by dividing the individual alert recommendations that were followed by the total alert instructions evoked. A recommendation was followed if there was a change in the underlying treatment management consistent with the individual instruction, generally within a two-hour period following the alert message. |
| Timeliness – Did the CDS alert tool reduce time to detect ARDS? | Percentage of clinicians reporting that alert decreased time to detection of ARDS |
| *CDS=Clinician Decision Support; ARDS=acute respiratory distress syndrome; LPV= Lung protective ventilation* | |
