# Supplementary material for: An alert tool to promote lung protective ventilation for possible acute respiratory distress syndrome
Source: JAMIA Open. 2022 Jul 8;5(2):ooac050. doi: 10.1093/jamiaopen/ooac050 (PMC9263532; doi:10.1093/jamiaopen/ooac050)
Supplement: ooac050_Supplementary_Data [file ooac050_supplementary_data.zip › supplemental_table_3_R1.docx]

| Supplemental Table 3. Survey participant demographics | | | | | | | |
| --- | --- | --- | --- | --- | --- | --- | --- |
| Characteristic |  | Unique clinicians surveyed | Response count (n) | Response rate | P-value for response rate difference | Using an automated alert fits with the way I like to work* | Using an automated alert fits with the way I like to work |
| Overall | Total | 158 | 53 | 34% |  | 0.79 | 0.69 |
| Role | Physicians | 93 | 35 | 38% | 0.19 | 0.77 | 0.68 |
|  | APPs | 65 | 18 | 28% |  | 0.83 | 0.70 |
| Sex | Female | 57 | 19 | 33% | 0.89 | 0.74 | 0.58 |
|  | Male | 101 | 34 | 34% |  | 0.82 | 0.74 |
| Self-reported years of experience | 0-5 | NA | 17 | NA | NA | 0.71 | 0.58 |
|  | 6-10 | NA | 10 | NA |  | 0.80 | 0.75 |
|  | 11-15 | NA | 7 | NA |  | 0.71 | 0.60 |
|  | >15 | NA | 19 | NA |  | 0.89 | 0.80 |
| Hospital location | A | 80 | 27 | 34% | <.001 | 0.70 | 0.60 |
|  | B | 17 | 8 | 47% |  | 1.00 | 1.00 |
|  | C | 16 | 5 | 31% |  | 0.60 | 0.60 |
|  | D | 19 | 2 | 11% |  | 1.00 | 1.00 |
|  | E | 17 | 7 | 41% |  | 0.86 | 0.75 |
|  | F | 6 | 4 | 67% |  | 1.00 | 1.00 |
| Survey type | Received alert | 53 | 22 | 42% | 0.13 | 0.73 | 0.60 |
|  | Did not receive alert | 105 | 31 | 30% |  | 0.84 | 0.75 |
| Notes: APP=Advanced practice providers; NA=data not available; *strongly agree, agree or indifferent. Significance (p<.05). | | | | | | | |
|  | | | | | | | |
