# Supplementary material for: An alert tool to promote lung protective ventilation for possible acute respiratory distress syndrome
Source: JAMIA Open. 2022 Jul 8;5(2):ooac050. doi: 10.1093/jamiaopen/ooac050 (PMC9263532; doi:10.1093/jamiaopen/ooac050)
Supplement: ooac050_Supplementary_Data [file ooac050_supplementary_data.zip › supplemental_table_4_R1.docx]

| Supplemental Table 4. Probability of disease presence given possible ARDS detection result (n=71) | | | | |
| --- | --- | --- | --- | --- |
|  | | Disease presence | | |
|  |  | Positive | Negative | Total |
| Possible ARDS detection result | Positive | 34 | 21 | 55 |
|  | Negative | 5 | 11 | 16 |
|  | Total | 39 | 32 | 71 |
|  | | | | |
| Measure | | Result | 95% CI Lower Limit | 95% CI Upper Limit |
| Prevalence | | .55 | .43 | .67 |
| Sensitivity | | .87 | .73 | .96 |
| Specificity | | .34 | .19 | .53 |
| Positive predictive value | | .62 | .48 | .75 |
| Negative predictive value | | .69 | .41 | .89 |
| Accuracy | | .63 | .51 | .75 |
| False positive rate | | .64 | .45 | .80 |
| Area under ROC | | .61 | .47 | .74 |
| ROC: receiver operating curve | | | | |
